# Suicide: Incidence or Prevalence? Comments on Hernández-Alvarado et al. Increase in Suicide Rates by Hanging in the Population of Tabasco, Mexico between 2003 and 2012. *Int. J. Environ. Res. Public Health* 2016, *13*, 552

**DOI:** 10.3390/ijerph13070671

**Published:** 2016-07-01

**Authors:** Julián Alfredo Fernández-Niño

**Affiliations:** Departamento de Salud Pública, Escuela de Medicina, Facultad de Salud, Universidad Industrial de Santander, Bucaramanga 680002, Colombia; jafernan@uis.edu.co

I recently reviewed the paper published in this journal by Hernández-Alvarado et al., titled “Increase in Suicide Rates by Hanging in the Population of Tabasco, Mexico between 2003 and 2012” [1], and I noticed that the epidemiological concept “prevalence” is not correctly used.

In the discussion the authors wrote: “However, this difference could be explained considering the fact that Tabasco State in Mexico has a historic higher prevalence of suicide than the rest of the country. However, the suicide prevalence remained unchanged in females” [1].

In that context, they refer to the observed changes in the rates of completed suicides in Tabasco State and the rest of Mexico. However, I consider that suicide is actually an excellent example of an incident event, which is never “prevalent”.

According to the National Institute of Mental Health, “prevalence” is defined as the “proportion of a population who have (or had) a specific characteristic in a given time period—in medicine, typically an illness, a condition, or a risk factor such as depression or smoking” [2]. In the same sense, the classic book of *Modern Epidemiology* by Rothman et al. defines the prevalence of a disease as “the proportion of the population with the disease at the specified time” [3]. Some authors also discriminate between “point prevalence”, “prevalence of period”, and “lifetime prevalence” [2,4].

First of all, I think that prevalence is not useful to measure the occurrence of an event that ceases to exist immediately. This is the case of completed suicide or any death because there is no longer anyone alive who has (or had) the event. The term “point prevalence” would be absurd, or at least inefficient, in describing this phenomenon, given that it would only include people who are committing suicide at a “given” time [2,3,4]. The terms “period prevalence” and “lifetime prevalence” do not work either because nobody retains the condition once it happens [3].

The solid fact is that a completed suicide obviously does not prevail over time. So, there are no “prevalent” suicides because they constitute deaths, and I think there is an obvious reason why we do not utilize the term “prevalence” to refer to deaths (the occurrence of death is defined as mortality). We can understand it better if we think about the term proposed by Rothman, “prevalence pool”, and we consider that “a person who dies with or from the state is removed from prevalence pool, consequently, death decreases prevalence” [3]. The problem is that when we talk about completed suicides, they are deaths themselves, and there is no “prevalence pool” or the pool is always empty; hence, there is nothing that qualifies as the “prevalence” of any kind of death.

Of course, we could use “prevalence” to refer to “suicide ideation” [5], due to the fact that ideation could be studied in cross-sectional studies and could be maintained through long periods of time in some people. Although suicide ideation is not always present all the time, except in some severe episodes of major depression, I think that we can use the terms point prevalence, period prevalence or lifetime prevalence for suicide ideation, as some authors have done it [5], in order to identify people with any suicide ideation at one specific point, at a specified period or at any time in their life, respectively.

In addition, we could estimate the lifetime prevalence or even the period prevalence for at least one suicide attempt at all in life, or in a specified period. However, definitely when we are analyzing the changes of the suicide rate or in general when we refer to completed suicides, we should not use the concept of “prevalence”. For completed suicide, we can describe its incidence, incidence rates, or the completed suicide rate [5].

This letter seeks the proper use of epidemiological terms in scientific publications and I hope my comments are useful for future papers on this topic.
